# Optimization of traction parameters for lumbar scoliosis

**DOI:** 10.1186/s12891-024-07583-y

**Published:** 2024-06-17

**Authors:** Wei He, Jia-Long Li, Jia-Yu Wang, Da He, Kai Song

**Affiliations:** 1 Mechanical and Vehicle Engineering, Hunan University, 2 Lushan South Road, Yuelu District, Changsha, 410082 China; 2 Spine of Department, Beijing Jishuitan Hospital, Capital Medical University, Beijing, 100035 China; 3 Department of Respiratory and Critical Care Medicine, the Second Xiangya Hospital, Central South University, Changsha, 410011 Hunan China

**Keywords:** Lumbar scoliosis, Finite element, Traction, Orthogonal experiment, ANOVA

## Abstract

**Background:**

Scoliosis is a high incidence disease that endangers the physical and mental health of adolescents. Traction therapy, as a conservative treatment plan, is helpful to improve the recovery speed of patients by studying the influence of different traction factors on the therapeutic effect.

**Methods:**

Based on the thin layer CT data of the lumbar spine of a 16-year-old patient with scoliosis, Mimics21.0 was used to extract the 3D digital model, and Geomagic Wrap2021 was used to perform the smooth surface. After that, SolidWorks was used to manually construct the structures, such as the intervertebral disc, and Ansys17.0 was used to add constraints, ligaments, and other features. Three-factor ANOVA was carried out after an orthogonal experiment that considered traction mode, traction angle, and traction force was finished.

**Results:**

① A three-dimensional biomechanical model of lumbar scoliosis was created. ② The model’s correctness was confirmed by comparing it to the corpse and other finite element models, as well as by verifying it under a range of working settings. ③ Traction force (*P* = 0.000), traction angle (*P* = 0.000), the interaction between traction force and traction angle (*P* = 0.000), and the interaction between traction mode and traction angle (*P* = 0.045) were all significant. ④ The interaction between traction force and traction angle has the most significant effect on Cobb, and traction with a certain angle is better than traditional axial traction. ⑤ Traction mode is not significant, but the interaction between traction mode and traction angle is significant.

**Conclusions:**

A certain angle of traction can aid in improving outcomes and the traction force can be suitably decreased in the clinical formulation of the traction plan. The uniformity of correcting effect is more favorable when higher fixation techniques like positive suspension or traction bed traction are used, as opposed to overhanging traction.

**Supplementary Information:**

The online version contains supplementary material available at 10.1186/s12891-024-07583-y.

## Background

Adolescent idiopathic scoliosis (AIS) is one of the conditions that endangers teenagers’ and children’s physical and mental health. The study found that the incidence of AIS is 5%, with a greater proportion of female patients than male patients [1]. Surgery for scoliosis is challenging and dangerous. It is recommended for patients with severe disease progression (Cobb > 40°) or serious complications; most of the disease is mild to moderate (Cobb < 40°), and the symptoms are mild, and the developing patients will be treated with conservative treatment such as wearing braces, gymnastics training, and respiratory training, which have been proven to have a certain effect on delaying the progression of the disease. Traction therapy is one form of conservative treatment that primarily corrects the patient’s bent vertebral body by applying axial traction to the patient’s spine. Traction therapy is also used in patients who need surgery, with the purpose of improving the flexibility of the patient’s spine before surgery, improving the success rate of surgery and reducing pain. Clinically, there are two types of traction therapy: suspension traction and horizontal traction [2]. With suspension traction, the patient uses their own body weight as a source of traction, which is managed by tightening their muscles. There’s also the traction approach, which involves tugging the patient from the ankle to perform a handstand. Some clinical tests have demonstrated that this traction approach is superior to positive suspension traction. Horizontal traction involves the patient lying flat on a traction bed, usually with the chest stabilized, and mechanical or manual traction is applied.

Joao Fialho proposed a mathematical model of scoliosis by establishing a mathematical model, and studied the determination of the patient’s personalized traction force and direction in robotic traction therapy, so as to achieve a personalized traction plan for each patient [3]. By comparing the human body’s trunk extension strength and flexion/extension ratio before and after suspension traction, Sung-Hak Cho et al. discovered that suspension traction helped to restore the neuromuscular control of the erector spinae muscle [4]. Tsung-min et al. discovered that suspending patients on a special horizontal bar and manipulating them might ease muscle spasms, adhesions, and lessen lumbar disc herniation [5]. Preoperative halo-gravity traction could provide partial correction, which could help eliminate aggressive treatments, enhance preoperative pulmonary function, and lessen neurologic complications [6]. Applying axial traction to patients may not be fully effective in treating the affected lumbar spine. In some studies, using traction at a certain angle is more beneficial for correcting scoliosis. There hasn’t been any research that simultaneously takes the traction mode, traction force, traction angle, and their interaction to enhance the traction effect into account when choosing the traction scheme. Using finite element analysis and a three-dimensional model of lumbar scoliosis, this paper offers a theoretical foundation for the creation of traction strategies in clinical practice.

## Methods

The subject of this study was a 16-year-old male patient with idiopathic scoliosis who weighed 60 kg and stood 170 cm tall. A thin-section CT scan of the whole spine was performed to obtain CT data in dcm format.

### Model building

**Fig. 1:**
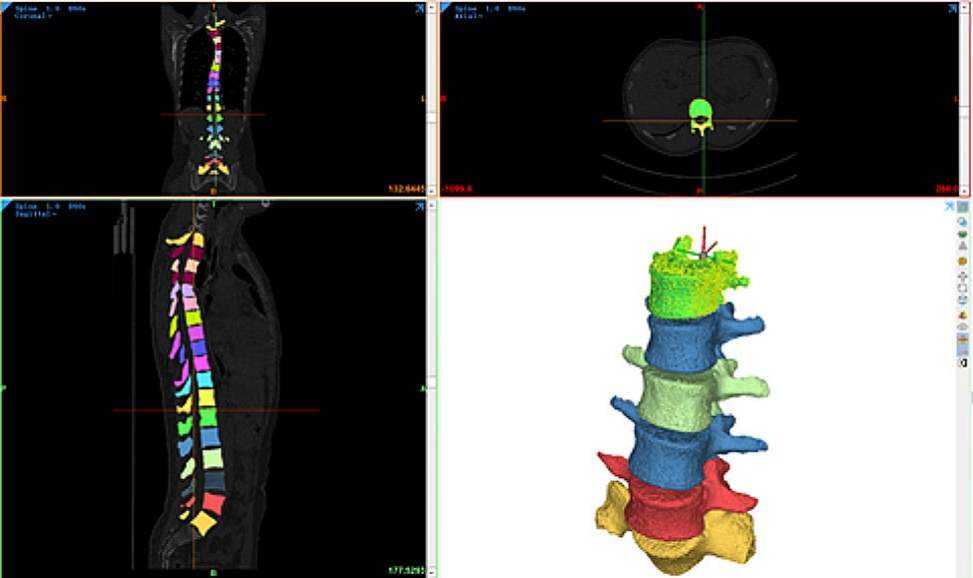
3D digital model extracted based on CT

A three-dimensional finite element model of a patient’s lumbar spine with scoliosis was created using the fundamentals of human anatomy. The lumbar vertebral body, intervertebral disc, endplate, articular cartilage, ligaments, and other soft tissues and human muscles were all incorporated in the model of the lumbar spine. The patient’s end vertebras were L2 and L4, and the Cobb angle was 10.65°. The patient’s raw data was imported in DICOM format into Mimics21.0 (Materialise Interactive Medical Image Control System, Materialize NV, Belgium). The dynamic region growing command only acquired the spine’s bone structure by modifying the software’s threshold. To create a 3D model of each lumbar vertebra, the software’s built-in segmentation and region growth commands were used to segment each vertebral body using a different color and fill the voids. Finally, each vertebral body segment was exported in STL format, as shown in Fig. 1.

Each lumbar spine’s STL file was loaded using Geomagic Wrap2021 (Geomagic Inc., Cary, NC, USA); the model’s exterior was then smoothed using sandpaper, rapid smoothing, relaxing, eliminating nails, and redrawing the mesh. The contour line was manually drawn to create the surface piece after the mesh doctor reviewed the pass, and the surface was then fitted to create the solid. The outside, 2 mm thick cortical bone and the inner, cancellous bone typically make up the vertebral body in an anatomical structure. Thus, in Geomagic, the cancellous bone is obtained by shifting the smooth vertebral body inward by 2 mm, and the solid is generated using the fitting surface. The cancellous and cortical bones were exported in STP format.

**Fig. 2:**
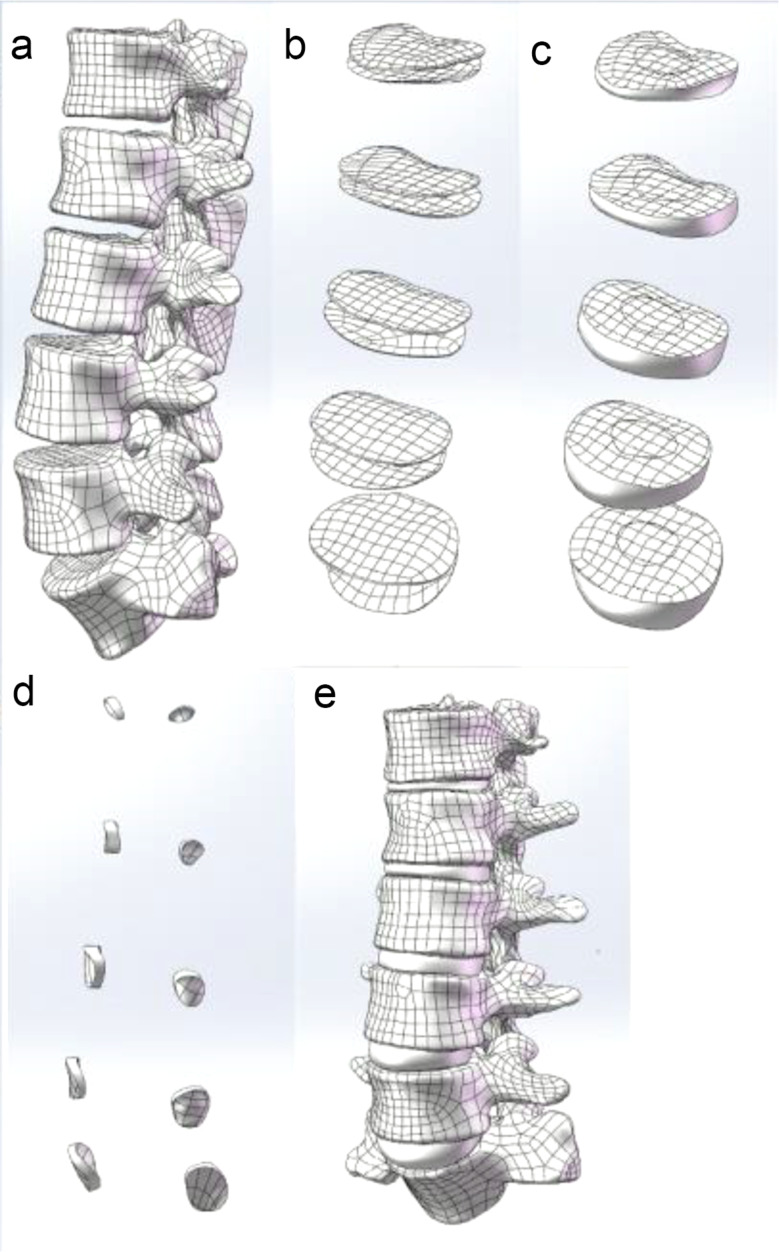
The vertebral body after smoothing (**a**). The reconstructed endplate (**b**). Reconstructed nucleus pulposus and annulus fibrosus (**c**). Reconstructed articular cartilage (**d**). The reconstructed lumbar spine model (**e**)

Since it is difficult for software to extract the lumbar spine’s intervertebral discs directly, manual creation is employed instead. Each vertebral body’s STP file was imported into SolidWorks2017 (Dassault Systèmes, Waltham, MA, USA), and the coincide with the origin command was used to match the vertebral body. The boss was stretched to create the facet articular cartilage, and the boss was lofted to create the disc. Segmentation produces the end-plate, annulus fibrosus, and nucleus pulposus via isometric surfaces on the disc. The volume of nucleus pulposus accounts for 30-40% of the intervertebral disc volume. Finally, it is exported as an SLDPRT file, as illustrated in Fig. 2.

Ansys17.0 (Ansys Inc., Technology Drive, Canonsburg., PA, USA) was used to import the established lumbar SLDPRT file. Based on the simplified model, the material was chosen as isotropic elastic. Refer to the published literature to define the material properties of bone, as shown in Table 1 [7–9].

**Table 1:** Material parameter settings for each structure of the trunk

Type of tissues	Young’s modulus(Mpa)	Poisson’s ratio
Cortical bone	12,000	0.3
Cancellous bone	1000	0.2
Annulus fibrosus	4.2	0.4
Nucleus pulposus	1	0.49
Endplate Cartilage	5000 10.5	0.25 0.45

**Fig. 3:**
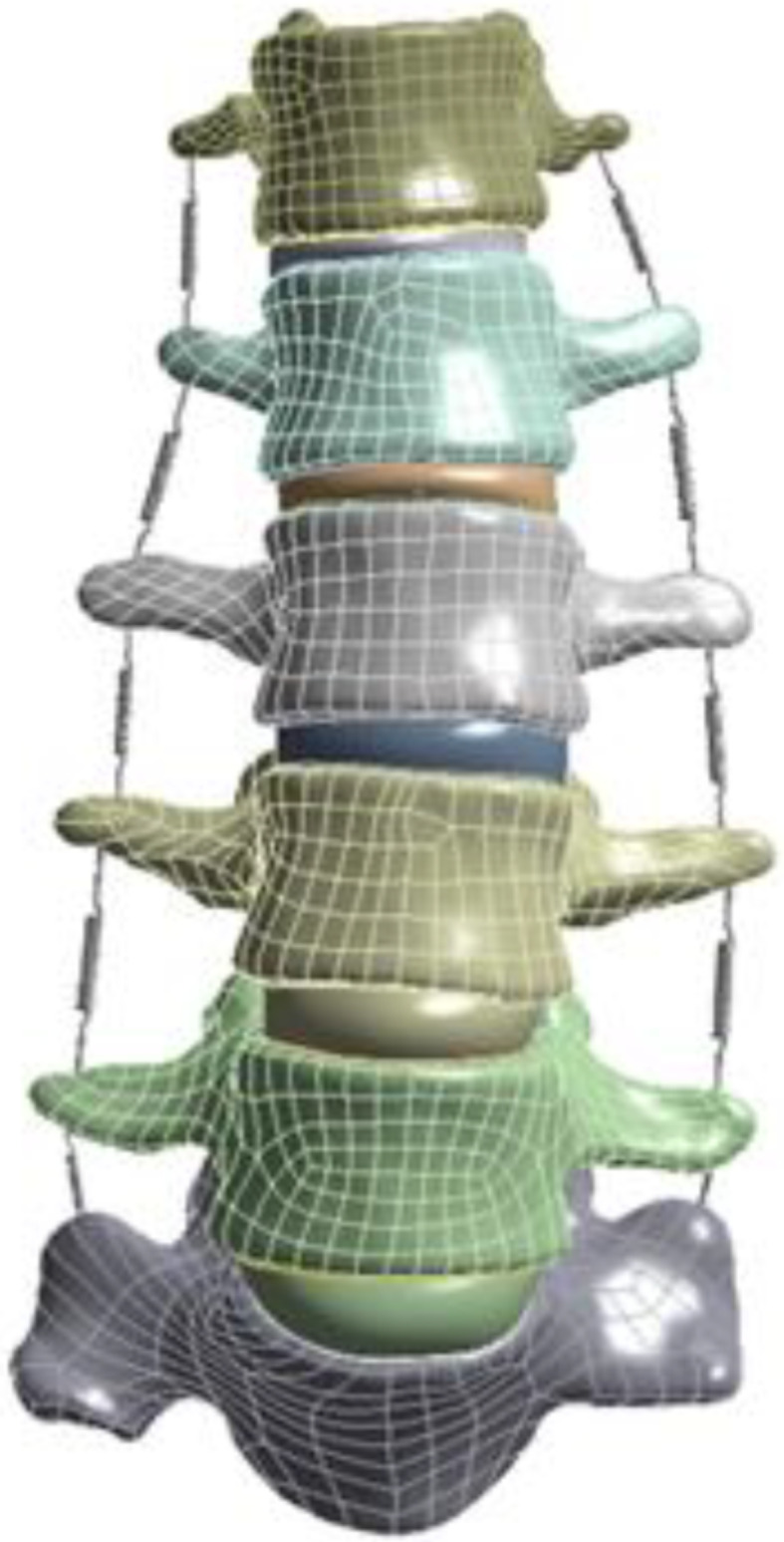
Model of scoliosis after adding ligaments

The anterior longitudinal ligament, posterior longitudinal ligament, supraspinous ligament, ligamentum flavum, and intertransverse ligament were among the ligaments between the vertebral bodies that Ansys simulated using springs. To define the ligament’s characteristics, consult the pertinent materials. For the material properties, see Table 2 [10–12]. The model once the ligament is established is shown in Fig. 3. On the coronal plane, the tilt angle of each vertebral body was measured, and the two vertebral bodies with the largest tilt toward the depression of scoliosis were found, and then the Cobb angle of lumbar spine was calculated.

**Table 2:** Ligament material properties

Structure	element type	stiffness (*N*·mm-1)
Anterior longitudinal ligament	spring	8.74
Posterior longitudinal ligament	spring	5.83
Intertransverse ligament	spring	0.19
Ligamentum flavum	spring	15.83
Interspinous ligament	spring	10.85
Supraspinous ligament	spring	2.39

**Fig. 4:**
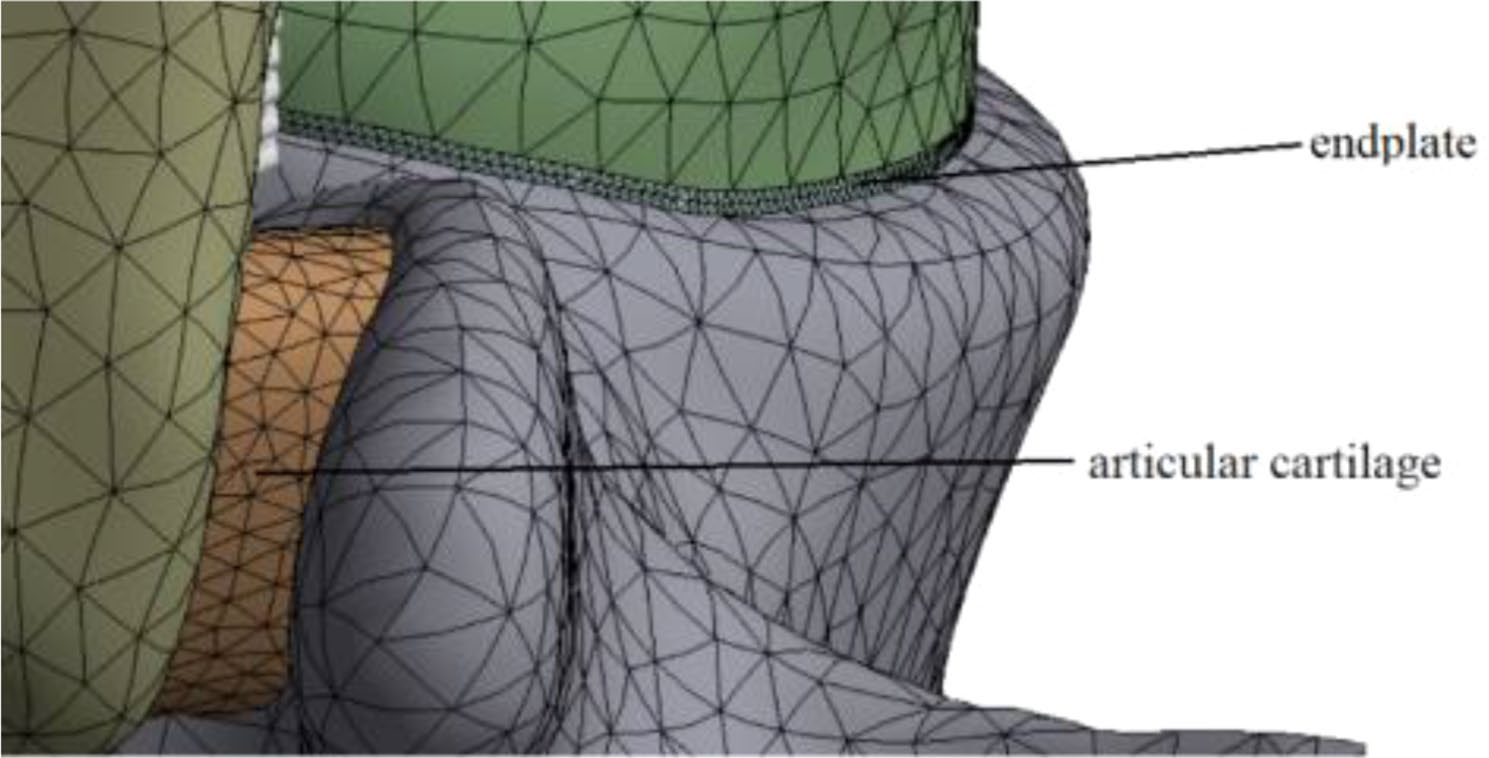
Mesh generation

In this model, there were two contact modes: ①Binding restraints were set between cortical bone and cancellous bone, cortical bone and endplate, endplate and annulus fibrosus, nucleus pulposus, annulus fibrosus and nucleus pulposus, vertebral body and the upper surface of articular cartilage; ②The vertebral body and the articular cartilage on the other side were set as non-separation constraints, and the normal displacement could not occur between them, but the movement between the contact surfaces was allowed. In order to ensure the accuracy of the later finite element calculation, three mesh sizes were used in this model: ①The vertebral body, annulus fibrosus and nucleus pulposus were used 2 mm mesh; ②1 mm mesh was used for facet articular cartilage; ③0.3 mm mesh was used for the endplate. See Fig. 4.

### Validation of the model

**Fig. 5:**
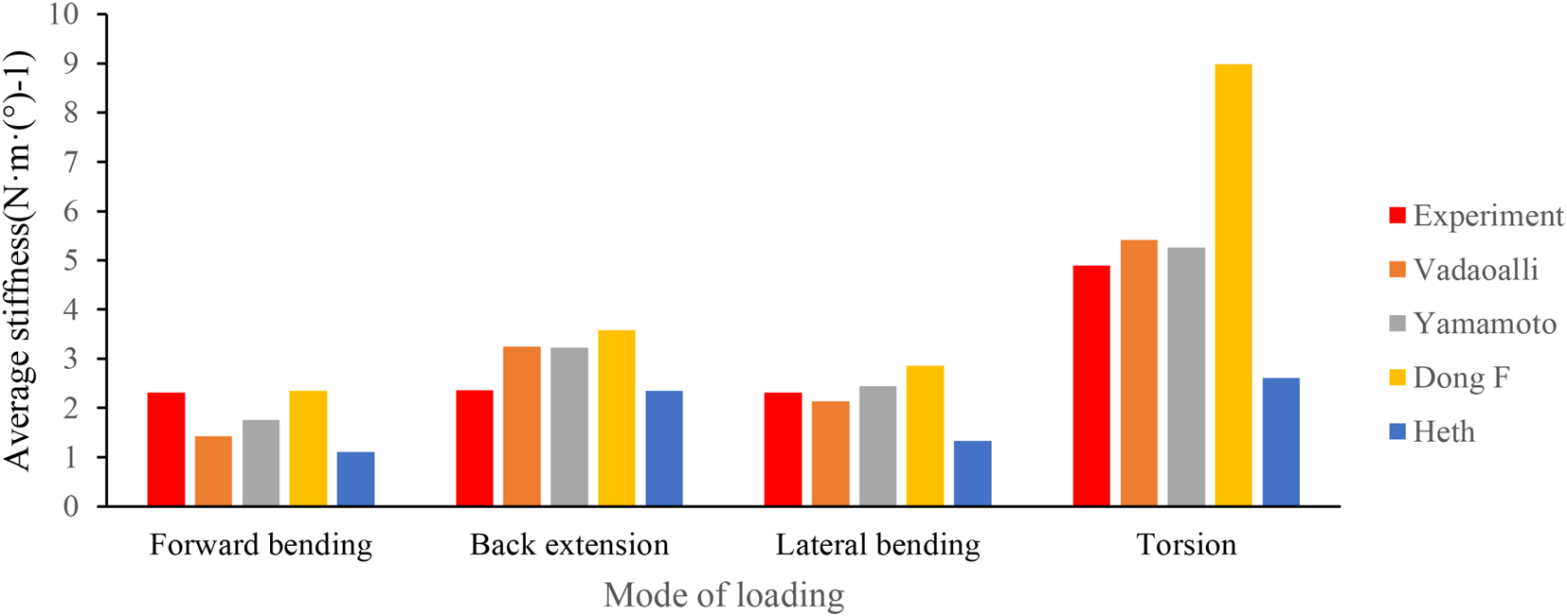
Comparison of average stiffness of finite element models

By contrasting the model’s average stiffness with that of other finite element models and the cadaveric body, the suggested finite element model’s validity was confirmed. The ratio of the torque applied to the vertebral body to the angular displacement in the torque’s direction was defined as the average stiffness. The average stiffness of this finite element model of scoliosis was calculated by applying a torque of 10 N·m under the same boundary conditions. This model is essentially consistent with the experimental results of Vadaoalli (L1-L2), Yamamoto (L1-L5), Dong F(FSU), and Heth (L2-S1) [13–15]. This suggests that the model is acceptable for finite element analysis, as illustrated in Fig. 5.

### Experimental design

The human body’s left and right directions were identified as X in the original CT scanning coordinate system, and the body’s right direction, pointing left, was identified as the X-axis forward. The human body’s anteroposterior orientation was identified as Y, and the Y axis forward denotes the front pointing in the direction of the back. The human body’s up-down direction was discovered to be Z, and S1 pointing to L1 was the Z-axis forward orientation. The orientation follows the law of the right-hand spiral.

**Fig. 6:**
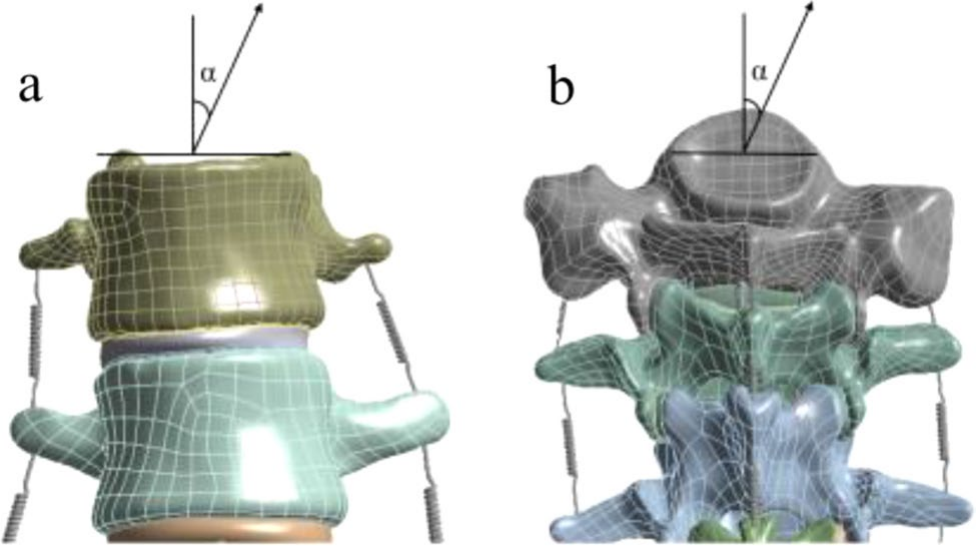
The traction force and traction angle under the lower fixation (**a**). The traction force and traction angle under the upper fixation (**b**)

According to Joao Fialho in robotic traction devices, the maximum traction for each patient in a course of treatment is 40% of the patient’s body weight [3]. In general, the degree of treatment is intended to cause excessive correction of the scoliosis spine, that is, to change the tilt of the vertebral body. Make sure the maximum traction is 240 N and the step size is set to 5%. Conventional axial traction is not effective on the diseased lumbar spine, so variable angle traction is applied in the same direction as the lumbar scoliosis. The diseased lumbar spine bends sideways in the positive X direction and rotates the direction of traction along the positive Y axis. When the traction angle is too large, the tangential component is too large to damage the lumbar spine. Based on relevant studies, the maximum angle is set at 35° and the step length is 5°. Traction methods are divided into two types:

① Fix the six degrees of freedom on the upper surface of L1, and traction is applied to the lower surface of S1 (upper fixation) to simulate bed traction;②Fix the six degrees of freedom on the lower surface of S1, and the traction force is applied to the upper surface of L1 (lower fixation) to simulate overhanging traction. Definitions of different traction force and traction angle are shown in Fig. 6.

Orthogonal experiment is a commonly used experimental method, which can effectively reduce the number of experiments. This experiment adopts a three-factor orthogonal experiment, and the specific experimental parameters and levels are shown in Table 3. The orthogonal array design of L32.4.8.8.1 is selected. To meet the level and factor requirements of this experimental design, the quasi-level method was used.

**Table 3:** Traction parameters and their levels

Symbol	Traction Parameters	Unit	Level1	Level2	Level3	Level4	Level5	Level6	Level7	Level8
A	Traction mode		Upper fixation				Lower fixation			
B	Traction force	N	60		120		180		240	
C	Traction angle	°	0	5	10	15	20	25	30	35

Cobb angle is a commonly used indicator to evaluate the severity of scoliosis in clinical practice. The smaller the angle is, the milder the symptoms are. Cobb is used as the only indicator.

In order to fully consider the interaction between the two factors, on the basis of 32 groups of orthogonal experiments, 12 groups of experiments were added, and a total of 44 experiments were done to meet the requirements of degrees of freedom.

## Results

### Finite element simulation

The original Cobb angle of the scoliosis patient was 10.65°. The parameters in Table 4 were used for the test, and the results obtained through Ansys simulation analysis were shown in Table 4.

**Table 4:** Experimental results for Cobb using the L32.4.8.8.1(The 4-level factors have eight, and the 8-level factors have one orthogonal table) orthogonal experiment

Exp.No(Combination)	Cobb(°)	Exp.No(Combination)	Cobb(°)	Exp.No(Combination)	Cobb(°)
1(A1B1C1)	9.9	16(A2B3C5)	9.78	31(A2B2C2)	9.51
2(A1B3C2)	8.75	17(A1B2C5)	6.67	32(A2B4C1)	9.8
3(A1B4C3)	6.95	18(A1B4C6)	17.82	33(A1B3C5)	10.11
4(A1B2C4)	7.58	19(A1B3C7)	14.54	34(A1B3C3)	7.51
5(A1B4C5)	14.69	20(A1B1C8)	7.61	35(A1B2C2)	9.5
6(A1B2C6)	7.74	21(A1B3C1)	10.45	36(A1B4C1)	10.14
7(A1B1C7)	8.01	22(A1B1C2)	8.14	37(A1B3C6)	12.74
8(A1B3C8)	18.23	23(A1B2C3)	8.41	38(A1B1C4)	9.27
9(A2B1C4)	9.31	24(A1B4C4)	10.84	39(A2B4C4)	10.32
10(A2B3C3)	6.89	25(A2B2C8)	10.68	40(A2B3C5)	7.05
11(A2B4C2)	7.77	26(A2B4C7)	20.39	41(A2B3C2)	8.77
12(A2B2C1)	10.41	27(A2B3C6)	12.49	42(A1B3C3)	8.66
13(A2B4C8)	23.17	28(A2B1C5)	9.09	43(A2B4C6)	17.35
14(A2B2C7)	11.55	29(A2B3C4)	8.19	44(A2B3C1)	9.93
15(A2B1C6)	8.95	30(A2B1C3)	9.48		

### Range analysis

Table 5 shows the results of Cobb angle calculated by range analysis based on the first 32 sets of orthogonal experimental data. k represents the average Cobb angle of each factor at the same level, and R represents the influence range of each factor on the experimental results within its value range. The larger R is, the more significant the influence of the level change of the factor on the experimental results is, and the more critical the factor is.

**Table 5:** Range analysis for cobb angle

	A		B		C
k_A1_	10.40	k_B1_	8.81	k_C1_	10.14
				k_C2_	8.54
		k_B2_	9.07	k_C3_	7.93
				k_C4_	8.98
k_A2_	11.09	k_B3_	11.17	k_C5_	10.06
				k_C6_	11.75
		k_B4_	13.93	k_C7_	13.62
				k_C8_	14.92
R_A_	0.69	R_B_	5.12	R_C_	6.99

According to the table, the order of the influence of each traction parameter on Cobb angle is as follows: Traction angle (C) > Traction force (B) > Traction mode (A).

### Variance analysis

Three-factor analysis of variance was used to study the relationship between traction mode, force and angle on Cobb. The calculation results are shown in Table 6.

**Table 6:** ANOVA analysis for Cobb

Source	Sum of Squares	Degree of Freedom	Mean Square	F	*p*
Intercept	4332.471	1	4332.471	100082.744	0.000**
A	0.029	1	0.029	0.659	0.476
B	127.956	3	42.652	985.286	0.000**
C	165.127	7	23.59	544.933	0.000**
A*B	0.284	3	0.095	2.187	0.269
A*C	2.911	7	0.416	9.607	0.045*
B*C	205.833	21	9.802	226.423	0.000**
Residual	0.13	3	0.043		

* *p* < 0.05 ** *p* < 0.01

As can be seen from the table, traction mode does not show significance, indicating that traction mode does not have a difference relationship with Cobb. The traction force and traction angle are significant, and the main effect exists, which will have a different relationship with Cobb. There are significant effects between traction mode and traction force, and between traction force and traction angle, indicating that there are interaction effects between them. The main order of influence of each traction factor on Cobb is as follows: Interaction of traction force and traction angle (B × C) > Traction angle (C) > Traction force(B) > Interaction of traction mode and traction angle (A × C) > Interaction of traction mode and traction force (A × B) > Traction mode (A). Therefore, in the process of formulating the traction scheme, the traction force and traction angle should be considered first and the interaction between them should be carefully selected. The traction mode does not require excessive attention.

### Interaction effect analysis

Interaction effect analysis helps to understand the circumstances in which the interaction between two factors makes a difference to Cobb.

**Fig. 7:**
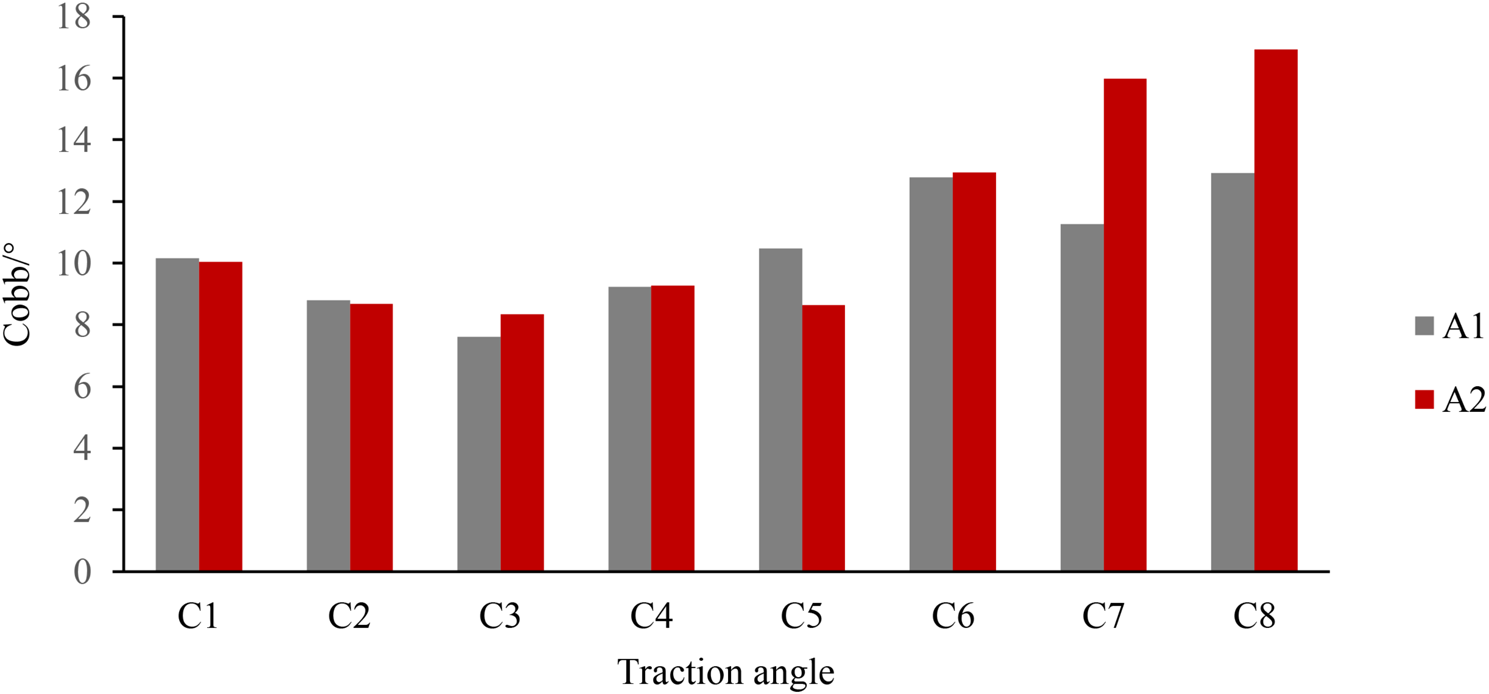
Comparison of mean values of traction mode and traction angle

As can be seen from Fig. 7, under both traction modes, the minimum Cobb can be achieved at a traction angle of 10°. Under the upper fixation mode, the difference of traction effect of different angles is smaller than that of the lower fixation mode. In the lower fixed mode, it is easier to obtain the opposite correction effect, so choosing the upper fixed traction mode is conducive to reducing the probability of producing the opposite correction effect.

**Fig. 8:**
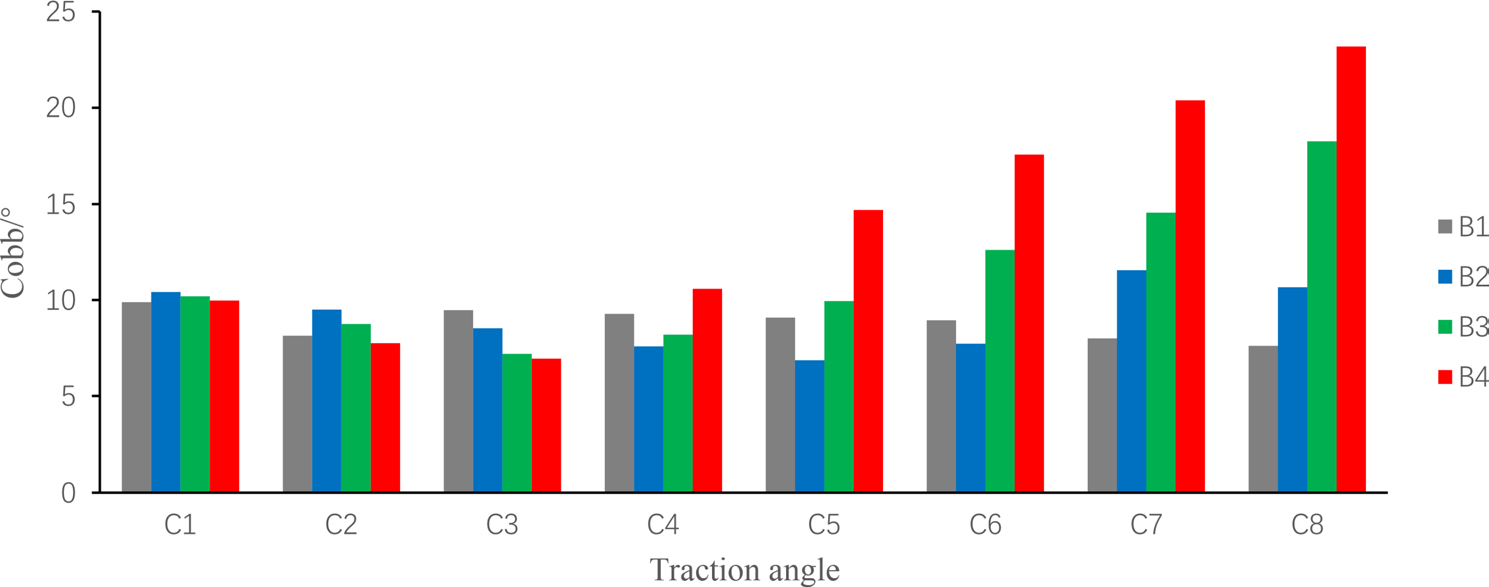
Comparison of traction angle and mean traction force

As can be seen from Fig. 8, under the condition of small traction angle, all kinds of traction forces can achieve a certain degree of correction effect, and traction at a certain angle can achieve better effects than traditional axial traction. The correction effect produced by a 120 N and 20° traction scheme is not very different from that produced by a 240 N and 10° traction scheme. With the increase of traction angle, the difference of correction effect between different traction forces increases. After the traction angle exceeds 15°, the reverse correction effect begins to appear. Therefore, it is necessary to strictly determine the interaction between the traction angle and the traction force in the process of formulating the traction scheme.

## Discussion

### Features of this model

Finite element method has been widely used in the study of human biomechanics [16, 17]. Based on reverse engineering and finite element simulation, this study established a three-dimensional finite element model of diseased lumbar vertebra according to CT data of patients with lateral curvature. The model included ligament, disc and other structures, and made reference to strictly demonstrated material properties. High precision can complete more accurate simulation. Under the action of the corresponding torque, the average stiffness of the model is verified and its validity is determined by comparing with various public data.

### Research significance and clinical value

Adolescent idiopathic scoliosis is the Cobb angle on the coronal surface before puberty or bone maturity > 10°, a common disease accompanied by vertebral rotation but without organic lesions, has become the fifth most common disease in adolescents worldwide after abnormal vision, obesity, phimosis and psychosocial disorders. The base of scoliosis patients in the world is growing fast, and the disease progresses rapidly before the bone is immature. If it is not controlled, the deterioration of the disease will have serious consequences such as affecting lung function. Clinically, traction therapy can effectively improve the lung function of patients, increase the flexibility of patients’ ligaments, and reduce the degree of deformity [18]. Known factors affecting the effect of traction therapy include traction force, traction time, traction angle, treatment time, patient posture during treatment, etc [19]. . Therefore, all factors affecting the effect of traction therapy should be studied so as to make traction program more effectively in clinic, speed up rehabilitation and reduce the occurrence of complications.

X-ray photography in traction is also an effective method to predict postoperative correction [20]. Most traction usually provides traction in only one direction, making it difficult to predict the outcome of the procedure. In the study, it was also proposed to further accurately predict postoperative outcomes by accurately controlling longitudinal traction force and lateral lateral thrust for better performance flexibility [21]. In clinical practice, it is common to use as much traction as possible within the tolerance of the patient, and as tolerance increases, so does the traction. In this study, when the maximum 240 N traction force was applied, the Cobb angle first decreased and then increased with the increase of the traction angle. It can be considered that the lateral component of the traction force increased with the increase of the traction angle, resulting in excessive overturning torque and the opposite effect.

Spinal traction is also a time-honored method for the treatment of disc herniation, degenerative disc disease and joint dysfunction [22]. Traction mainly reduces lower back pain by increasing disc height and creating a gap between the facet joints. Farajpour H studied the effects of traction at different angles on the lumbar ligaments and found that the optimal angle should be determined based on the patient’s disc height decline, weight, and height [23]. Similar to scoliosis traction, a herniated disc traction at an angle can also achieve better correction results. For patients with scoliosis, different traction forces, traction angles and traction methods have different effects on different ligaments between different vertebral bodies, and the behavior of ligaments is also different, which is an important factor leading to the differences in effects. Therefore, it is important to determine the traction factors according to the ligament that needs to be stretched.

### Limitations of the study

In this experiment, only one lumbar spine with scoliosis was modeled, and the influence of factors such as the type of scoliosis cannot be excluded. There is a certain degree of simplification in the modeling process, no muscles are added, and the force and displacement of the pelvis and lumbar spine are not considered. This study only studied the traction factors from the behavior of each vertebral body of the lumbar spine (Cobb), but did not study the internal law of scoliosis from the stress and strain.

## Conclusion

The method proposed in this study is effective in predicting the outcome of traction for scoliosis. Clinically, we can appropriately reduce the traction force on the original traction plan, adopt a small-angle traction plan, and adopt a unified upper-fixed traction method, so as to achieve better results. In addition, CT or MRI images can be used to establish a personalized model of scoliosis patients. The results of this study can reduce the time of simulation analysis with different traction parameters and provide appropriate personalized traction parameters for treatment.

### Electronic supplementary material

Below is the link to the electronic supplementary material.

Supplementary Material 1

## Data Availability

The data underlying this article are available in the article and in itsonline supplementary material.
